# Hepatolithiasis caused by right hepatic artery branches forming an arterial ring compressing the common hepatic duct

**DOI:** 10.1093/jscr/rjac492

**Published:** 2022-10-31

**Authors:** Yifan Wang, Benjamin M Motz, Matthew S Strand, John B Martinie, Dionisios Vrochides, Erin Baker, David A Iannitti

**Affiliations:** Division of Hepato-Pancreato-Biliary Surgery, Department of Surgery, Carolinas Medical Center, Charlotte, NC, USA; Division of Hepato-Pancreato-Biliary Surgery, Department of Surgery, Carolinas Medical Center, Charlotte, NC, USA; Division of Hepato-Pancreato-Biliary Surgery, Department of Surgery, Carolinas Medical Center, Charlotte, NC, USA; Division of Hepato-Pancreato-Biliary Surgery, Department of Surgery, Carolinas Medical Center, Charlotte, NC, USA; Division of Hepato-Pancreato-Biliary Surgery, Department of Surgery, Carolinas Medical Center, Charlotte, NC, USA; Division of Hepato-Pancreato-Biliary Surgery, Department of Surgery, Carolinas Medical Center, Charlotte, NC, USA; Division of Hepato-Pancreato-Biliary Surgery, Department of Surgery, Carolinas Medical Center, Charlotte, NC, USA

## Abstract

Anatomic variations of the hepatic artery do not usually cause biliary obstruction. We present a 51-year-old male who developed biliary obstruction and hepatolithiasis due to extrinsic compression of the common hepatic duct (CHD) by an arterial ring formed by the anterior and posterior branches of the right hepatic artery. We performed a surgical bile duct exploration and used intraoperative direct cholangioscopy to guide clearance of hepatolithiasis. Herein, we review the existing literature on CHD compression caused by topographical variants of the hepatic artery and discuss diagnostic and treatment strategies.

## INTRODUCTION

The right hepatic artery (RHA) typically crosses posterior to the common hepatic duct (CHD), and subsequently divides into an anterior and a posterior sectoral branch. Anatomic variants of the hepatic artery are seen in ~20% of patients [1, 2]. Although recognition of these variants is important for surgical planning, their presence is generally considered to be of no physiologic significance. In 1984, Tsuchiya *et al.* described a topographical variant where the RHA crossed anterior to the CHD and led to biliary obstruction [3]. Here, we present a patient who had early branching of the RHA, with the right anterior and right posterior arteries encircling and compressing the CHD resulting in proximal hepatolithiasis.

## CASE REPORT

**Figure 1:**
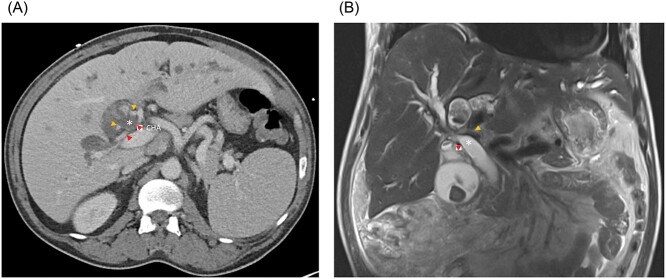
(**A**) Axial CT and (**B**) coronal MRI imaging showing the right anterior (orange arrowheads) and right posterior (red arrowheads) hepatic arteries encircling the common hepatic duct (white asterisk). CHA, common hepatic artery.

A 51-year-old male presented with abdominal pain, jaundice and fever. His medical history was significant for type 2 diabetes mellitus and dyslipidemia. He had no history of liver disease, pancreatitis or hepatobiliary surgery. He had a cholestatic elevation of liver enzymes with a total bilirubin of 17.6 mg/dL and alkaline phosphatase of 508 IU/L. Computed tomography (CT) showed intrahepatic biliary dilatation and large stones within the left intrahepatic bile duct (Fig. 1A). The common bile duct was dilated to 18 mm and there was a stone at the ampulla. Magnetic resonance imaging (MRI) demonstrated bilateral hepatolithiasis and a focal CHD stricture near its bifurcation (Fig. 1B).

At endoscopic retrograde cholangiopancreatography (ERCP), a sphincterotomy was performed and the stone at the ampulla was extracted. The stricture near the CHD bifurcation was dilated with a balloon catheter. Visualization of the intrahepatic ducts was limited by the extent of hepatolithiasis. A plastic biliary stent was placed in the left hepatic duct. The carbohydrate antigen (CA 19–9) and carcinoembryonic antigen levels were within normal range. Since the hepatolithiasis could not be cleared endoscopically, surgical common bile duct exploration was performed.

**Figure 2:**
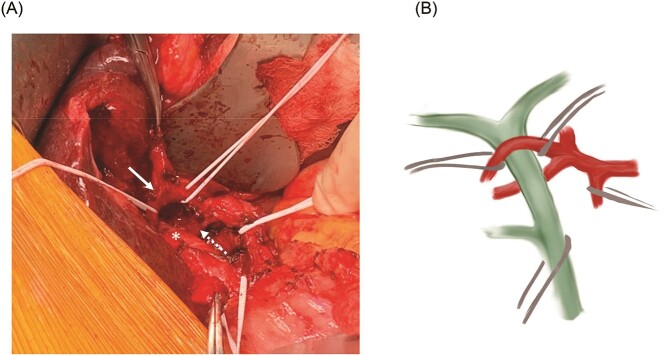
(**A**) Relationship between the common hepatic duct (asterisk) and the mobilized anterior (solid arrow) and posterior (dotted arrow) RHA branches. (**B**) Schematic representation of the RHA variant anatomy.

At laparotomy, we identified an early bifurcation of the RHA into its anterior and posterior branches, immediately distal to the branching of the left hepatic artery. The right anterior artery crossed anterior to the CHD, whereas the right posterior artery coursed posterior to the CHD (Fig. 1A and B). These arterial branches were densely adherent to the CHD, and were circumferentially constricting the CHD. After completing a cholecystectomy, we performed an arterial divestment, dissecting in the periadventitial plane along the course of the RHA, to release the anterior and posterior RHA branches from the CHD (Fig. 2A and B). Intraoperative ultrasound confirmed extensive hepatolithiasis, predominantly in the left hepatic duct. We transected the CHD 1 cm distal to its bifurcation and transposed the anterior RHA branch posterior to the CHD. We used Spyglass™ Discover Digital Catheter (Boston Scientific, Natick, MA) direct cholangioscopy to guide clearance of the hepatolithiasis. Intrahepatic stones were extracted using stone forceps and biliary Fogarty catheters. Cholangioscopy revealed no intrahepatic strictures and normal appearance of the biliary epithelium. The CHD was reconstructed in an end-to-end fashion over bilateral plastic biliary stents. Histological evaluation of the gallbladder revealed chronic cholecystitis and a segment 4B liver biopsy showed changes consistent with chronic cholestasis. The patient had an uneventful postoperative course and has remained asymptomatic after 3 months of follow-up.

## DISCUSSION

This case illustrates a rare phenomenon of CHD compression within an arterial ring formed by the anterior and posterior sectoral branches of an early branching RHA. Prolonged biliary stasis caused by this extrinsic compression may have contributed to the development of hepatolithiasis.

In 80% of patients, the RHA crosses posterior to the CHD in the hepatic hilum [1, 2]. Tsuchiya *et al.* described the first cases of hepatolithiasis and CHD compression caused by a RHA crossing anterior to the CHD^3^. They coined this entity the ‘right hepatic artery compression syndrome’. In 79 additional patients with hepatolithiasis, 2 of 11 patients with an anterior RHA had CHD stenosis at the level where the RHA crossed the CHD. In contrast, none of the 68 patients with a conventional posterior crossing RHA had CHD compression.

Since this initial report, 10 additional cases of CHD compression caused by topographical variants of the hepatic artery have been described (Table 1) [4–11]. The most common variant was a RHA crossing anterior to the CHD. The majority of patients underwent surgery for bile duct exploration and bilioenteric drainage, or to release the artery from the CHD. Both surgical approaches achieved a high rate of symptom resolution. If the variant artery is mobilized to release its impingement on the CHD, a bilioenteric drainage procedure may not be necessary. One advantage of performing a bile duct exploration alone without a bilioenteric anastomosis is that it preserves the ability to perform ERCP in native anatomy. In recent years, there have been reports describing purely endoscopic management using ERCP or cholangioscopy to clear intrahepatic stones. However, endolumenal therapies do not resolve the underlying CHD compression, and there is a paucity of data on their long-term outcomes. Furthermore, large intrahepatic stones may not be amenable to endolumenal extraction. Here, we suggest that direct cholangioscopy is a useful intraoperative tool to guide complete clearance of the hepatolithiasis. Moreover, to facilitate future ERCP interventions, we favored CHD transection to position the CHD anterior to the RHA branches, with primary end-to-end reconstruction of the CHD, rather than a bilioenteric drainage procedure.

**Table 1:** Published cases of common hepatic duct compression by hepatic arterial variants

**Author (Country)**	**Year**	**Number of patients**	**Age**	**Sex**	**Arterial variant**	**Treatment**	**Outcome**
Tsuchiya *et al.* (Japan) [3]	1984	2	59	F	Anterior RHA	CBDE + H-J	No recurrence × 2 years
			57	F	Anterior RHA	Left liver resection + H-J	Postoperative mortality
Goldberg *et al.* (USA) [4]	1988	1	30	F	CHA branches forming ring	Release of vascular ring + T-tube	No recurrence × 20 months
Chung *et al.* (South Korea) [5]	1994	1	39	F	Anterior RHA	Release of RHA + cholecystectomy + T-tube	No recurrence × 6 months
Kullman *et al.* (Sweden) [6]	2000	1	55	M	Anterior PHA	Release of PHA + cholecystectomy + IOC	No recurrence × 15 months
Miyashita *et al.* (Japan) [7]	2005	1	55	M	Posterior RHA	CBD resection + H-J	No recurrence × 1 year
Bilanovic *et al.* (Serbia) [8]	2011	1	68	F	PHA forming ring	Release of PHA + CBDE + cholecystectomy + T-tube	No recurrence × 5 years
Eshtiaghpour *et al.* (USA) [9]	2011	1	54	M	Anterior RHA	ERCP + IOC + biliary stent	No recurrence × 3 months
Mendes *et al.* (Belgium) [10]	2014	1	52	F	Anterior RHA	CBDE + H-J	No recurrence × 24 months
Bove *et al.* (Italy) [11]	2019	3	65 (mean)	M	Anterior RHA	ERCP + stone extraction	No recurrence × 12 months

F, female; M, male; RHA, right hepatic artery; PHA, proper hepatic artery; CBDE, common bile duct exploration; H-J, hepatico-jejunostomy; ERCP, endoscopic retrograde cholangiopancreatography; IOC, intraoperative cholangioscopy

Establishing a preoperative diagnosis of this condition is challenging. Cross-sectional imaging can help delineate the topographical relationships between the CHD and the hepatic arterial vasculature. An anterior crossing RHA or RHA branches encircling the CHD should raise suspicion for ‘RHA compression syndrome’, particularly if the CHD demonstrates a focal caliber change at that location. ERCP may visualize a CHD stricture, but has limited ability to differentiate between benign and malignant strictures. To this end, cholangioscopy allows for direct visualization of the biliary epithelium. Furthermore, direct cholangioscopic visualization of a pulsatile compression of the CHD by the RHA may support this diagnosis [9].

In summary, extrinsic compression of the CHD by topographical variants of the hepatic artery should be considered in the differential diagnosis of extrahepatic biliary obstruction. Although release of the arterial impingement may be sufficient to relieve the biliary obstruction, there are not sufficient data to determine whether bilioenteric drainage offers a long-term benefit.
